# Towards model-based control of Parkinson's disease

**DOI:** 10.1098/rsta.2010.0050

**Published:** 2010-05-13

**Authors:** Steven J. Schiff

**Affiliations:** Center for Neural Engineering, Departments of Neurosurgery, Engineering Science and Mechanics, and Physics, Pennsylvania State University, University Park, PA 16802, USA

**Keywords:** Parkinson’s disease, control theory, computational neuroscience, deep brain stimulation, Kalman filter

## Abstract

Modern model-based control theory has led to transformative improvements in our ability to track the nonlinear dynamics of systems that we observe, and to engineer control systems of unprecedented efficacy. In parallel with these developments, our ability to build computational models to embody our expanding knowledge of the biophysics of neurons and their networks is maturing at a rapid rate. In the treatment of human dynamical disease, our employment of deep brain stimulators for the treatment of Parkinson’s disease is gaining increasing acceptance. Thus, the confluence of these three developments—control theory, computational neuroscience and deep brain stimulation—offers a unique opportunity to create novel approaches to the treatment of this disease. This paper explores the relevant state of the art of science, medicine and engineering, and proposes a strategy for model-based control of Parkinson’s disease. We present a set of preliminary calculations employing basal ganglia computational models, structured within an unscented Kalman filter for tracking observations and prescribing control. Based upon these findings, we will offer suggestions for future research and development.

## 1. Model-based control and neuroscience

This morning, you awoke to a not unreasonable weather forecast. The most recent aeroplane you flew on may well have autolanded by a control system without pilot intervention. Both of these seemingly disparate activities are examples of the revolution created by modern control theory to observe (weather) and control (airframes) complex systems. In both of these cases, a computational model, embodying our *a priori* knowledge of the system at hand, was the key to the success.

It seems incredible that the tremendous body of skill and knowledge of model-based control engineering has had so little impact on modern medicine. Dynamical diseases are diseases characterized by the operation of a biological control system in a region of physiological parameters that produces pathological behaviour (Mackey & Glass 1977). Dynamical diseases of the brain are those where the symptoms are created by abnormal patterns of activity within neuronal networks (Milton & Jung 2003). The timing is now propitious to propose fusing control theory with neural stimulation for the treatment of dynamical brain disease.

There were some good reasons for this lack of intersection of these disciplines in the past. Modern control engineering uses computational models of a system (the airframe’s equations of motion, or the convection physics of the atmosphere in the case of weather prediction) to perform the assimilation of observable data, the reconstruction of unobservable variables in the system state, the estimation of parameters (constant or slowly changing variables) and the short-term prediction of the system state. This sequence is then followed by the next iteration of data assimilation, and so forth. Biology and medicine were cut out of interacting with such control engineering until computational models were developed of sufficient applicability to warrant use. In 2010, our sophistication in computational biology is certainly now at hand. But the other issue impeding the application of control theory to biology was the nature of biological dynamics—most are floridly nonlinear.

Modern (model-based) control engineering had its roots in the control of linear (or linearizable) systems. In parallel with the development of the early US space programme, Kalman (1960) devised a filter, which for linear systems is a maximum-likelihood estimator, that gives optimal tracking of the system state and optimal calculation of control signals to change such states. The dual theorems of *observability* and *reachability* for such systems have been considered one of the most useful developments in mathematics of the twentieth century (Casti 2000). Observability and reachability theorems essentially state that if you can observe a system’s state variables, you can optimally control it (to reach a given state).

Kalman’s original filter was fast, but had its limits. Nonlinear models were handled for several decades by linearizing equations about the operating points of the system state using an extended Kalman filter (EKF). Such linearization is notoriously unreliable in many systems, often dramatically shown in the simple conversion of polar to Cartesian coordinates in range-locating systems (sines and cosines do not linearize well). Biology often regulates its systems far removed from the simple homeostasis of the early twentieth century work of Walter Cannon (Cannon 1932)—witness the pulsatile secretion of parathormone (Schiff & Deftos 1995), the contractility of the heart or the firing of a neuronal action potential. Prior to the late 1990s, the only solution to such serious nonlinearities would be to employ Monte Carlo techniques (such as particle filters) to iterate an ensemble of system states by one-by-one iterating each point in an estimated distribution of states through the nonlinear equations (see Spiller *et al.* (2008) for detailed discussion of particle filtering versus the EKF in nonlinear systems). Particle filters have been explored to some degree in biological systems, but their inherent inefficiency renders them, at present, almost entirely inapplicable to real-time observation and control.

In the late 1990s, Julier & Uhlman (1997*a*,*b*) published a highly efficient method of parameterizing state uncertainty for nonlinear systems that they termed the unscented Kalman filter (UKF). Within a decade, it has become a mainstay of the robotics scientist (Thrun *et al.* 2005). In parallel, from within an almost completely separate literature, numerical meteorology developed their ensemble Kalman filter (EnKF) and its variants (Evensen 1994; Evensen & van Leeuwen 2000). Both UKFs and EnKFs do the same thing. They permit us to fly an aeroplane in real time, and to iterate huge convection models of the atmosphere several times per day, using nonlinear models with unprecedented efficiency and accuracy.

In 2004, towards the end of an obscure physics paper, Voss *et al.* (2004) demonstrated that action-potential dynamics of single neurons might be amenable to tracking with such a nonlinear UKF.

We have now spent several years exploring the implications of the Voss *et al.* work. We first extended Voss’s approach to a framework for the analysis of spatio-temporal data from cortical voltage-sensitive-dye imaging experiments. We showed that such an approach was not only feasible, but that it was also robust to large amounts of measurement noise. By using an observer system run in parallel with the experimental system, we could very significantly reduce the energy required to control such a system with electrical feedback (Schiff & Sauer 2008). Voss, as did we, employed reduced simplified models of neurons (either Fitzhugh–Nagumo or Wilson–Cowan equations).

Would this strategy work with biophysically realistic models of neurons? We recently showed that not only could the foundational Hodgkin–Huxley ionic equations be incorporated into such a control framework, but also that a UKF strategy was rather striking in its ability to accurately reconstruct the entire set of Hodgkin–Huxley conductance and rate variables given only voltage measurements (Ullah & Schiff 2009). Our guess is that such success must be related to the intrinsic independence of ionic currents and time constants present in these equations, and of course in the real neurons upon which they are based—the symmetries (discussed in §8) are insufficient to impair the reconstruction.

Hodgkin and Huxley left out that these neuronal dynamics were intimately coupled to complex metabolic ion dynamics, both extracellularly and coupled to glia. We have recently worked out a comprehensive computational framework to account for the dynamics of potassium flux into and out of neurons and glia, as well as the effect of such flux dynamics on the excitability of neuronal networks (Cressman *et al.* 2009; Ullah *et al.* 2009). We demonstrated in our models that such ionic dynamics could account for major components of the phenomenology that we observe experimentally in the dynamics of epileptic seizures.

We then demonstrated that our control framework could readily incorporate such ionic dynamics as well. We showed a powerful way to perform dynamic clamp, using a more complete reconstruction of the cellular dynamics, rather than the more isolated conductance relationships customarily used (Ullah & Schiff 2009). We also showed how to potentially incorporate this ionic framework to modulate seizure dynamics (Ullah & Schiff 2009).

We recognize that variability is profound in biology, whether from the genome, proteome or species level (Kirschner & Gerhart 2005). Compounding the difficulties with variability, the complexity of all biological systems far surpasses the details in our models. All models are bad to some degree (even that of a simple pendulum), but our models of biological cell, network and organismal dynamics are terrible by comparison with, for instance, our models of airframes. We therefore began to develop a comprehensive strategy to directly deal with model inadequacy in such biological systems. We showed that we can, as part of our control frameworks, construct a locally optimized set of mean parameters that we term the *consensus set*. We have recently demonstrated our ability to track the dynamics from real spatio-temporal data from our brain experiments, using a local consensus set, and have shown that our tracking errors converge to unexpected accuracy (Sauer & Schiff 2009). Such a strategy can be broken up to local tessellations of networks when complexity demands this, and there are new rigorous EnKF tessellation methods in the numerical weather-prediction literature (Ott *et al.* 2004) ideal for such use.

The implications of the consensus set are that we are now able to use a model network of sufficient complexity and connectivity to accurately observe a real brain network despite uncertainties in cell dynamics and connection topology. Our short-term prediction accuracy appears sufficient for control. This is a completely different situation from the customary strategy of *model validation*—where one assumes that the model is correct, and that only the parameters need to be fitted. The identical situation was found in a recent physics application of local EnKFs to fluid dynamics—the best parameters to use in a simplified model are not the most physical, but in recognition of model inadequacy are the optimal ones to best track observed dynamics (Cornick *et al.* 2009). To be blunt, the best parameters to use for such tracking and control work can be (bio)physically meaningless. But the model dynamics are not meaningless. Our conjecture is that, for much of biology, seeking model validation in control scenarios may be the wrong goal. Seeking models of sufficient complexity to accurately emulate and track the dynamics of the state of a biological system may be a more relevant strategy.

This is not just a matter of making better control devices for biological systems. We often consider that if we simply amass sufficient computer hardware and software, we will be able to create large-scale models that would be faithful replicas of structures such as the brain. Were such a thing possible, we would have much trouble gaining insight into how it worked, but access to all relevant variables would be a valuable feature that we could never have in a real brain. Most important is the sheer mass of parts and components to model—this is a fundamental ‘limits to knowledge’ problem, as eloquently discussed by Scott (1995). All of us, whether modelling for an underpowered implantable device, or for the world’s most powerful supercomputer, need to deal with serious model inadequacy when faced with real biology and disease.

All control filters, whether linear Kalman or nonlinear UKF/EnKFs, are at their essence synchronization problems (Duane *et al.* 2006; Yang *et al.* 2006). That is, a suitable control filter will synchronize to the natural system it is observing when its performance is good. It is now well established that nonlinear systems can synchronize, and when not identical can nonlinearly synchronize—generalized synchronization (Schiff *et al.* 1996). Our techniques of establishing such synchronization often have made use of knowing the equations from the systems involved, except for a special case—when auxiliary systems are used. It turns out that, when identical model systems, with different initial conditions, are driven by the same signal from a potentially unknown system, at a certain level of driving they can ‘forget’ their initial conditions and synchronize (Pyragas 1996). If you perturb one of the driven systems, it exponentially dissipates its perturbation and re-synchronizes with the other driven system. This means that if we use biological models of sufficient complexity, and perturb one of the identical copies intermittently, we can both infer the presence of synchronization with the inadequately modelled biological system, and furthermore quantify the model inadequacy in real time. Such model inadequacy leads us to adjust our uncertainty in the tracking systems, through the so-called covariance inflation (Danforth & Yorke 2006; Yang *et al.* 2006). This gives us the ability to probe a complex biological system using synchronizing controllers—the closer we get to the underlying dynamics, the closer the synchronization.

All of the above suggests that we are at the verge of a transformation in our capabilities of tracking and controlling brain dynamics in health and disease. This paper will discuss the prospects for applying such knowledge to Parkinson’s disease. In §2, we will present an overview of Parkinson’s disease, in §3, discuss the networks of Parkinson’s disease, in §4, describe the thalamus, in §5, describe compounds that can cause a Parkinson’s disease-like state, in §6, discuss the dynamics of Parkinson’s disease networks, in §7, describe a deep brain stimulation (DBS) paradox, in §8, describe a reductionist approach to this paradox, in §9, discuss control cost functions, in §10, discuss fusing data with models, in §11, describe building a control framework for Parkinson’s disease dynamics and in §12, summarize and discuss future directions. The goal of this paper is to serve as a prelude to more comprehensive model-based control and experimental applications in the future.

## 2. Overview of Parkinson’s disease

**Figure 1.**
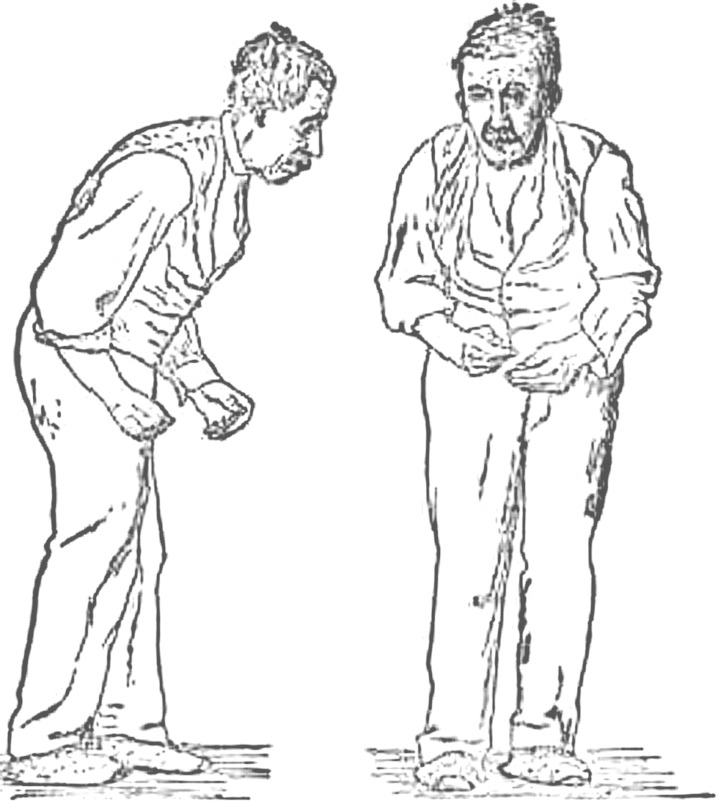
A well-marked case of this disease, as described by William Gowers. (Adapted from Gowers (1901).)

Parkinson’s disease was first described by James Parkinson in a monograph published in 1817 (Parkinson 1817). It is a degenerative neurological condition, and we attempt to digest the neurological manifestations of this disease into four key signs: tremor, rigidity, bradykinesia (slowness of movement) and postural instability. But a far better sense of the signs of this condition was captured by the short description by William Gowers in his 1901 textbook (Gowers 1901) that flanked his famous sketch of a typical patient walking (reproduced in figure 1)

… the aspect of the patient is very characteristic. The head is bent forward, and the expression of the face is anxious and fixed, unchanged by any play of emotion. The arms are slightly flexed at all joints from muscular rigidity, and (the hands especially) are in constant rhythmical movement, which continues when the limbs are at rest so far as the will is concerned. The tremor is usually more marked on one side than on the other. Voluntary movements are performed slowly and with little power. The patient often walks with short quick steps, leaning forward as if about to run.

(Gowers 1901, p. 639)

We have learned a considerable amount about the neurobiology of Parkinson’s disease since the nineteenth century, but we do not know how to prevent it, and all of our present-day treatments remain palliative (Lees *et al.* 2009). The discovery by Carlsson *et al.* (1957) that a precursor to dopamine in the brain could ameliorate the effects of dopamine depletion led to successful medical therapy of Parkinson’s disease with l-3,4-dihydroxyphenylalanine (l-DOPA). Early on in the disease, l-DOPA provides the chemical precursor to produce more of the waning dopamine neurotransmitter, but gradually there are fewer of these cells remaining that can benefit from such a boost in chemical processing. Furthermore, there are two side effects that patients find disturbing: (i) the symptoms that return during the wearing-off phase after taking a dose, producing more radical on–off swings in motor symptoms and (ii) the gradual development of involuntary movements termed *dyskinesias* (Schapira *et al.* 2009). Although drug therapy remains the first-line standard of treatment for patients with Parkinson’s disease, the long-term medical side effects of phamacological therapy have kept the surgical treatment options for pharmacologically intractable Parkinson’s disease alive.

There are three surgical lesion targets in the brain that have been found to reduce the symptomatology of Parkinson’s disease effectively: the ventral intermediate nucleus (VIM) of the thalamus, the internal segment of the globus pallidus (GPi) and the subthalamic nucleus (STN). Because patients with Parkinson’s disease generally require treatment on both sides of the brain, the efficacy of single-sided lesion treatment was often tempered by the complications of bilateral therapy. One never wants to lesion a brain symmetrically. So, lesions can be administered assymetrically. One is also hesitant to use too large a lesion at any one sitting, so there was a frequency of having to return to surgery to enlarge a lesion that was not effective enough.

Parkinson’s disease has become the disease most widely treated so far by DBS (Kringelbach *et al.* 2007). DBS appeared to be effective when used bilaterally, without the symmetrical lesion complications. In 1997, the US Food and Drug Administration approved the use of DBS in Parkinson’s disease. Stimulation of the same targets that were lesioned produced palliative effects (Koller *et al.* 1999). VIM lesions and stimulation were well characterized to reduce tremor, but although tremor is a hallmark of the disease, it is not the most disabling symptom for most patients. To better deal with the bradykinesia and rigidity, stimulation of the GPi and STN became the preferred targets. Although the STN is now the dominant DBS target, it is unclear whether the GPi, with a less burdensome set of cognitive side effects, might be better for some patients (Anderson *et al.* 2005). As our experience with DBS has progressed, a direct comparison of pharmacological versus DBS (bilateral GPi or STN stimulation) for Parkinson’s disease found DBS to have advantages in quality-of-life outcomes in comparison with pharmacological therapy, despite the risks inherent in surgical treatment (Deuschl *et al.* 2006; Weaver *et al.* 2009). Nevertheless, there remains interest in lesions, whose effectiveness versus pharmacological therapy has also been shown (Vitek *et al.* 2003), and whose long-term medical management and costs are considerably less than for patients who require lifelong maintenance of DBS systems (Blomstedt & Hariz 2006). An especially compelling study of bilateral subthalmotomy argues the rationale for lesions and demonstrates the apparent safety and risk assessment of symmetric STN lesions (Alvarez *et al.* 2005). The issue requires continued debate, as the majority of patients with Parkinson’s disease on the planet, and their healthcare systems, do not have the resources to consider DBS therapy.

The lesion debate notwithstanding, there is no more fruitful arena to consider a radical new approach to neural systems control than DBS technologies for Parkinson’s disease. Our present approach has been to focus on high-frequency stimulation (130 Hz) delivered in open loop without feedback sensing. Along with the rise of such empirical DBS therapy, we have developed increasingly sophisticated computational models of the fundamental networks involved in the pathophysiology of Parkinson’s disease. And we have developed increasingly sophisticated models of the physics of DBS stimulation to help us understand how DBS interacts with neurons. Let us examine this in detail.

## 3. The networks of Parkinson’s disease

A great deal of the brain, especially the regions beneath the cortex, is heavily involved with movement regulation. Such areas include the connected set of basal ganglia, portions of the thalamus and the cerebellum. The cerebellum may well contain as many neurons as the rest of the brain. Coordination and movement require a lot of mind.

**Figure 2.**
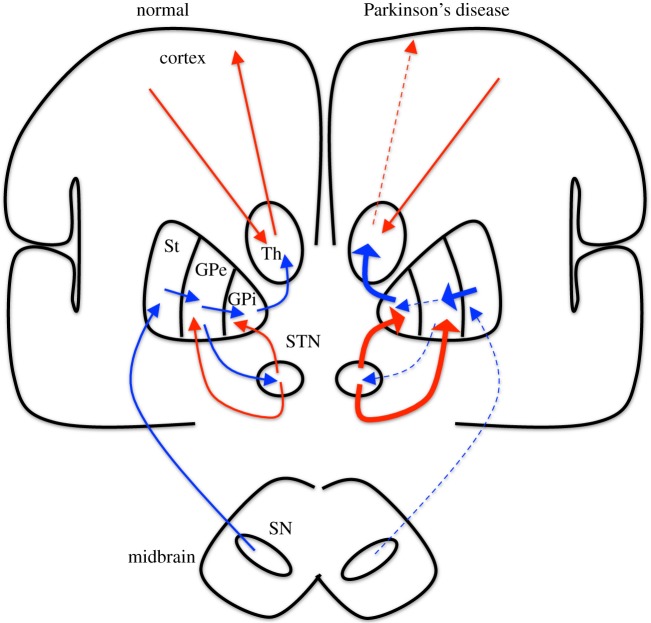
An extremely simplified schematic of network imbalance in Parkinson’s disease. Excitation in red and inhibition in blue. The contrast with normal in the Parkinson’s disease state is shown on the right, where thickened (thinned) lines indicate an increase (decrease) in excitation (red) or inhibition (blue). St, striatum; GPe, globus pallidus externa; GPi, globus pallidus interna; Th, Thalamus; STN, subthalamic nucleus; and SN, substantia nigra. I have made no distinction between indirect and direct pathways, and customized this for the purposes of the discussion within this paper. For a more complete and detailed description of this anatomy, see Obeso *et al.* (2008).

In Parkinson’s disease, there is degeneration of neurons that use dopamine as a neurotransmitter, which have their cell bodies in the substantia nigra at the upper edge of the midbrain. The decrease in neural output from the substantia nigra causes a disturbance in the network balance of excitation and inhibition, as schematized in figure 2. The result is a net increase in inhibition from the GPi to thalamus (for a much more detailed discussion of the circuitry, see Obeso *et al.* (2008)). But the lines and arrows in these static diagrams refer to average firing rate or activity, and do not reflect the dynamics that is critical to understand what is happening. In Parkinson’s disease, the inhibition to the thalamus becomes phasic and oscillates.

If, in figure 2, you place a thin probe and burn a small hole in the GPi or STN, the symptoms of tremor, bradykinesia and rigidity decrease.^1^ We have understood the improvement in symptoms from the schematic in that the excessive amount of inhibition streaming out of the GPi and into the thalamus will be decreased. If you instead place a thin electrode into these same structures, and stimulate at 130 Hz, you will get an almost identical decrease in the symptoms of tremor, bradykinesia and rigidity. And this paradox requires explanation.

## 4. The thalamus—its not a simple relay anymore

The thalamus (latin for *inner chamber*) is a walnut-sized piece of very-high-end real estate in the centre of the brain. We used to view it predominantly as a relay centre for things such as sensory information. The thalamus has a variety of relay stations re-synapsing touch, vibration, sound and visual information onto a fresh set of neurons coursing to their respective cortical targets (see schematic in Guillery & Sherman 2002). If substantial damage is caused to the thalamus, such as from a stroke, the brain will lose consciousnes (Posner & Plum 2007). Neuroscientists, evolutionary biologists, epileptologists and philosophers have always gravitated to the human cortex as the object of their fascination. But gradually, the reality of a cortex heavily interconnected with a series of relay loops to the deep thalamus has emerged, substantially complicating the older views (Guillery & Sherman 2002).

Transmission within the cortex tends to be slow, with local conductance velocities in the range of centimetres per second, in contrast to the fast long-range neural connections where many tens of metres per second are commonly observed. We think that the thalamus forms an essential transcortical relay system to help integrate information processing across cortical areas where local conduction speeds would otherwise render us far dimmer.

It is the reliability of this relaying of motor information through the thalamus that will be a focus of the following computational theory. Nevertheless, one must ask why, if there is such an intimate relationship between cortex and thalamus, is so much of the medical treatment focusing on the lesioning or stimulation of targets many inches into the brain? Recent clinical trials have explored this issue, but so far the results of these early efforts at superficial cortical stimulation have not been as effective as DBS or lesions (Gutiérrez *et al.* 2009).

## 5. The contribution of China White

The study of the dynamics of Parkinson’s disease was immeasurably helped by the seemingly inherent cravings of humans for narcotics. In the late 1970s, synthesizing heroin-like compounds such as 1-methyl-4-phenyl-4-proprionoxy-piperidine (MPPP) could produce a byproduct, perhaps with a bit too much heat or acid in the reaction, of the related 1-methyl-4-phenyl-1,2,3,6-tetrahydropyridine (MPTP). A solitary report of poisoning in this manner quietly appeared in Davis *et al.* (1979). By 1982, a clandestine drug chemist in California was selling this product under the name *China White*, until a bad batch started producing profound Parkinson’s disease symptoms in a group of young addicts (Langston *et al.* 1983). It was quickly discovered that this compound could produce the same symptoms in non-human primates (Burns *et al.* 1983). It was shown from both the human and animal data that MPTP was very selective in destroying the dopamine containing neurons in the pars compacta of the substantia nigra—the same substructure that prominently degenerated in human Parkinson’s disease. The symptoms and signs of the drug-induced and natural disease were nearly identical, and both diseases responded to treatment with dopamine precursor drugs (l-DOPA). The full story of these events is remarkable (Langston & Palfreman 1995; Factor & Weiner 2002).

The critical benefit from these events was the proof that loss of a small group of neurons in the substantia nigra could produce the triad of tremor, rigidity and bradykinesia. The primate animal model provided us with a way to dramatically increase both our knowledge of the electrophysiology of the neuronal networks involved and their potential electrical modulation, to the extent that the following can be described.

## 6. Dynamics of Parkinson’s networks

Prior to 2002, most models of Parkinson’s disease were displayed as static diagrams (as in figure 2). Nevertheless, the advent of the MPTP primate model (Raz *et al.* 2001; Wichmann & DeLong 2003), and increasingly the recording of neurons from human Parkinson’s patients during deep brain surgery (Magnin *et al.* 2000; Brown *et al.* 2001), revealed that the neurons within the Parkinsonian networks were strongly oscillatory (thalamus, GPi and the external segment of the globus pallidus, GPe). In a fundamental experiment, Plenz & Kital (1999) observed that if they mixed bits of the GPe and STN in tissue culture that the cells would spontaneously connect and generate oscillations.

In the schematic of the basal ganglia, there are fundamental features of *central pattern generators* seen in the connectivity of the GPe and STN. GPe cells inhibit each other as well as the STN. The STN excites the GPe. When STN cells are inhibited, they also demonstrate an exaggerated rebound excitation (Bevan *et al.* 2002). Any simple neuronal membrane will spike if released suddenly from an inhibitory input—*anode break excitation* (Hodgkin & Huxley 1952). Some of the neurons critical to Parkinsonian dynamics have additional currents that exaggerate such inhibition-induced rebound excitation.

**Figure 3.**
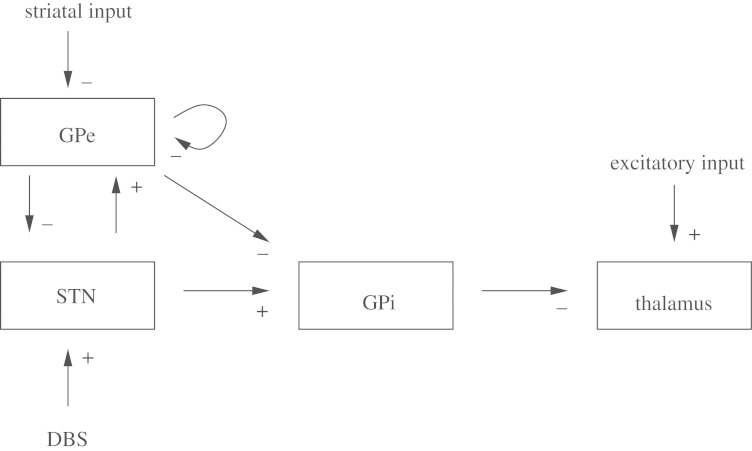
Schematic of rhythm generating structures in Parkinson’s disease. Striatal input refers to the outer segments of the basal ganglia that send input to these deeper segments. Excitatory input refers to sensorimotor input to the thalamus that needs to be relayed to cortex. Excitation, +; inhibition, −. (Adapted from Rubin & Terman (2004).)

 Terman *et al.* (2002) set out to explain these network effects on the basis of the biophysical properties of the individual neuronal types and their synaptic connectivity. They focused on the essence of what appeared to be the rhythm generating circuitry, which turns out to also be the targets for both lesioning and stimulation in surgical therapy (their schematic is reproduced in figure 3).

Applying typical experimental techniques in brain studies, we can record from single cells. But only in exceptional circumstances do we survey enough pairs of connected cells to develop strong characterizations about the details of the connectivity. There have been heroic experiments where investigators spent years carefully sticking micro-electrodes into pairs of neurons to directly sample such connectivity (Traub & Miles 1991), but such efforts remain rare. To make up for the paucity of connectivity data, Terman *et al.* (2002) explored a range of connectivities. Terman *et al.* (2002) had a great deal of intracellular data from networks from brain-slice experiments that preserved native network architecture and connectivity (see Hallworth & Bevan 2005 and references therein) to draw upon.

**Figure 4.**
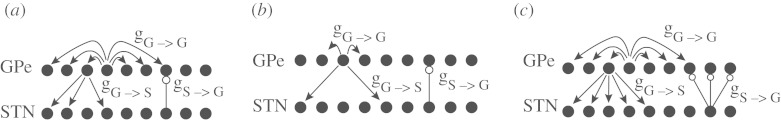
(*a*) Sparse random network. Each STN neuron excites a single random GPe cell, and each GPe neuron inhibits three random STN cells. GPe cells inhibit each other through all-to-all coupling. (*b*) Sparse structured network. Although more structured than the random sparse network of (*a*), it is designed to avoid direct reciprocal connections between STN and GPe cells. Each STN neuron excites the single closest GPe cell, and each GPe neuron inhibits two STN cells, skipping the three closest. GPe cells inhibit two immediate neighbouring GPe cells. (*c*) Tightly connected structured network. Each STN neuron excites three closest GPe cells, and each GPe neuron inhibits the five closest STN cells. GPe cells inhibit each other through all-to-all coupling. Spatially periodic boundary conditions are applied (the network wraps around on itself). (Adapted from Terman *et al.* (2002).)

Three types of network topologies were constructed by Terman *et al.* (2002): random and sparsely connected, structured and sparsely connected, and structured and tightly connected, as shown in figure 4.

To model the STN neurons, and bring out their rebound excitability, Terman *et al.* (2002) model the STN membrane current as a combination of
6.1
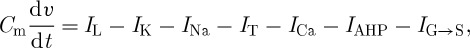

where *C*_m_ is membrane capacitance.

The first three currents (*I*_L_−*I*_K_−*I*_Na_) are the Hodgkin–Huxley leak, rectifying potassium and fast sodium that evolution elected to conserve from cephalopods to mammals (Hodgkin & Huxley 1952). In this case, these currents had parameters tailored to the mammalian neurons rather than the squid axon. *I*_G→S_ represents the current injected into these cells from the GPe synapses. And now three special currents will be described: *I*_AHP_, *I*_Ca_ and *I*_T_.

The afterhyperpolarization current, *I*_AHP_, is a potassium channel that opens in response to increasing amounts of intracellular calcium. While it is on, it prevents the cell from firing another spike, so it takes an important role in turning off excitation, as well as regulating firing frequency. It is a good way to create resonance-frequency ranges within which a cell would prefer to fire. And it is a good way to generate a pacemaker. It is prominent in motoneurons in the spinal cord, where the frequency of discharge must be matched to the characteristics of the muscle fibres they connect to (fast or slow) (Kernell *et al.* 1999). *I*_AHP_ is prominent in the suprachiasmatic nucleus, where it helps translate the molecular clock of our circadian rhythms into neuronal firing frequency (Cloues & Sather 2003). And *I*_AHP_ is important in the STN, helping to make the neurons more sensitive to inputs at motor frequencies (Bevan & Wilson 1999), and helping to create an oscillatory central pattern generator out of the network it is embedded within in the basal ganglia (Bevan *et al.* 2002).

The high-threshold calcium current, *I*_Ca_, is a representation of what are probably several high-threshold calcium channels in such cells (Song *et al.* 2000). High threshold means that they are activated with depolarization. Because the calcium Nernst reversal potential is very positive (even more positive than sodium), these channels in general support regenerative potentials (i.e. they boost depolarization already in progress). Bursting cells tend to have such channels.

The low-threshold calcium current, *I*_T_, is prominently seen in thalamic neurons where, following inhibition, such neurons rebound burst fire (McCormick & Bal 1997). This current activates upon hyperpolarization, and then deactivates more slowly than *I*_Na_ deactivates as the neuron depolarizes. As the reversal potential for calcium is positive, activating such a current gives the neuron a boost to bring its membrane potential away from rest and accentuate the tendency of any neuron to rebound a bit (as anode break excitation). *I*_T_ plays a role in the prominent post-inhibitory rebound spiking seen in STN cells (Bevan *et al.* 2002). Interestingly, the same T-type calcium currents play a role in the automaticity and pacemaker function in heart cells, and can play a role in certain cardiac arrhythmias (Vassort *et al.* 2006).

 Terman *et al.* (2002) used the same currents as equation (6.1) for modelling GPe neurons, but changed their proportions so that they better matched the firing properties seen in a variety of experimental studies.

In the sparse random network (figure 4*a*), the strength of connectivity (maximal synaptic conductance) was varied from the STN to GPe, and within the GPe to GPe network. Increasing the STN to GPe coupling produced a range of behaviours from sparse irregular firing, to episodic bursting, to continuous firing. The episodic regime was qualitatively similar to that reported in the classic study by DeLong (1971) for cells in the normal primate GPe. One of the counterintuitive features of increasing the GPe to GPe inhibition is that it can increase the spread of activity through rebound. In the modelling of Terman *et al.* (2002), the episodic burst firing is terminated by *I*_AHP_ as calcium is built up in GPe cells during the high-frequency firing episodes.

In the sparse structured network (figure 4*b*), there was more temporal clustering in the interactions among these cells, reflecting an increase in the topological spatial clustering of the neuronal connections.

In the tightly connected structured network (figure 4*c*), they also wrapped the network around on itself (periodic boundary conditions). This network generated waves that travelled. Initiating such waves required symmetry breaking in the STN-to-GPe and GPe-to-STN footprints. To form a solitary travelling wave, the GPe-to-GPe inhibitory footprint had to be spatially larger than the STN-to-GPe footprint (supporting a Turing instability; Murray 2003). Key here was that, for a wave to be propagated, the STN and GPe cells had to have structured footprints so as to orderly spread activity to cells ahead of the leading edge of the wave (Terman *et al.* 2002).

Travelling waves have been experimentally observed in thalamic slices, generating what are called spindle oscillations (Kim *et al.* 1995). Spindle waves have been related to sleep physiology, but the relationship to Parkinson’s disease is not presently clear. Nevertheless, whenever there is evidence of temporal oscillations in a neural network, it is worth considering what the spatial structure of those oscillations are (Huang *et al.* 2004). Especially when networks are sparsely connected (as opposed to all-to-all connected where there is no meaningful spatial structure), considering whether waves might underlie such rhythms is a reasonable question.

**Figure 5.**
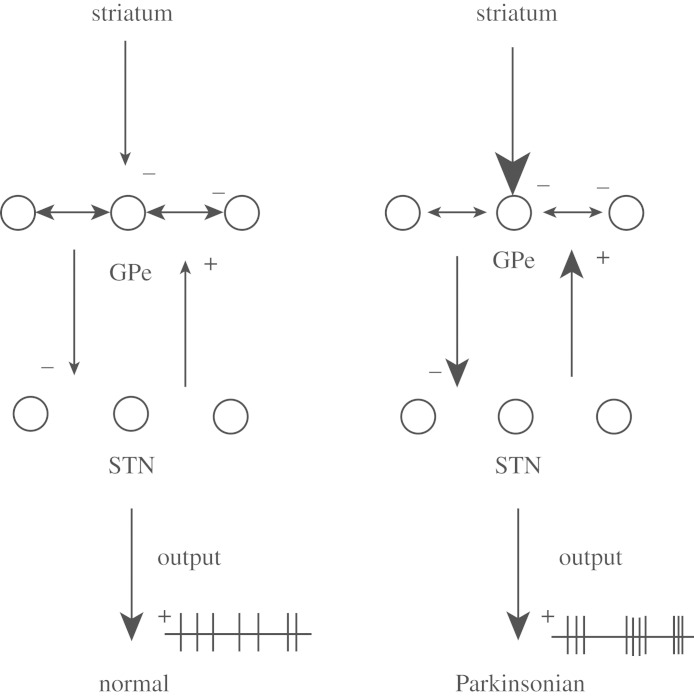
Schematic of Terman *et al.* (2002) suggesting how an increase in striatal input and decrease in GPe internal connections would generate the oscillations of a Parkinsonian state. (Adapted from Terman *et al.* (2002).)

Strong oscillations emerge in the GPe–STN network in Parkinson’s disease and in dopamine depletion in experimental animals. Nevertheless, there is a body of experimental evidence, in both human patients and MPTP primates (see Terman *et al.* 2002), that fails to find the sort of highly correlated and synchronized firing that would support the coherent waves predicted in the most structured networks of figure 4. The picture emerging from this work is that, among the more sparse networks, the conversion from normal to Parkinsonian dynamics fits well with the schematic in figure 5. This schematic illustrates that, following a loss of dopamine input to the striatum, a strengthening of striatal input to the GPe, perhaps with a concurrent weakening of recurring inhibitory connections within the GPe, could create a Parkinsonian state.

Now, go back to the standard firing-rate model of Parkinson’s disease from figure 2. In this static model, a *decrease* in inhibition to the striatum led to an increased activity of the STN, which increased excitation to the GPe and GPi, which increased the inhibition to the thalamus. No oscillations arise in the static model, in a disease whose hallmark are the oscillations both within and outside the brain. In the world view of Terman *et al.* (2002), the level of striatal input (inhibitory) is a major regulator of whether oscillations arise from within this network that is naturally wired and poised to oscillate. Increased striatal inhibition effectively strengthened the coupling from the STN to the GPe. Decreasing intra-GPe inhibition would promote clustered activity within the GPe. And inhibition, as is found in other dynamic phenomena in the nervous system such as seizures, plays multi-faceted and complex roles (Ziburkus *et al.* 2006; Mann & Mody 2008). Inhibition in neuronal systems, similar to inhibitory reactants in reaction diffusion systems, is crucial in organizing patterns into clustered as opposed to homogeneous patterns (Murray 2003).

## 7. The deep brain stimulation paradox

DBS of the GPi or STN has almost identical effects on the symptoms of Parkinson’s disease as do lesions of the GPi or STN. We have evidence of excessive activity of the GPi in Parkinson’s disease, but stimulation of the STN should further increase the GPi activity.

For several years, it was therefore assumed that stimulation at the DBS frequencies being used (typically 130 Hz) must have been suppressing activity within the nuclei. This was supported by data showing that recording in the vicinity of the cell bodies within the STN when stimulating the STN (Benazzouz *et al.* 2000), or recording within the GPi with the GPi stimulation (Boraud *et al.* 1996), demonstrated a decrease in apparent cell firing following nearby high-frequency stimulation. Helping to make further sense of this were the findings, in neurons different from the basal ganglia, that synaptic depression could occur at modest stimulation frequencies while the synapses ploddingly worked to repackage neurotransmitters within synaptic vesicles for release (Staley *et al.* 1998). These findings were all consistent in explaining why DBS had the same effects as ablative lesions. This assumption was unfortunately wrong.

In computational modelling, McIntyre *et al.* (2004) demonstrated that such DBS close to cell bodies and axons might preferentially initiate action potentials further out along the axons of such neurons than we have appreciated previously, and furthermore that the cell bodies might not reflect these action potentials. Such findings also remind one of the differing requirements in the curvature of a stimulating electrical potential field needed to initiate firing at the terminal end of a neuron process membrane, as opposed to the middle of a membrane such as an axon coursing near an electrode *en passage* (Warman *et al.* 1992). More recently, optical imaging has also confirmed that action potential initiation may be well beyond the axon hillock in contrast with what we have previously assumed (Bikson *et al.* 2004).

**Figure 6.**
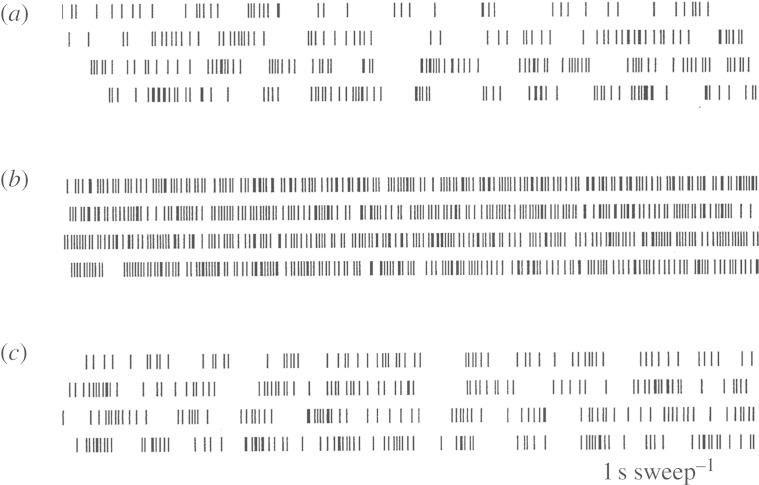
Demonstration that 130 Hz stimulation of the STN in an MPTP primate increased the GPi cell firing rates. (*a*) Pre-stimulation, (*b*) during 136 Hz stimulation and (*c*) post-stimulation. (Adapted from Hashimoto *et al.* (2003).)

The definitive experimental evidence that laid to rest the hypothesis that DBS worked by suppressing neuronal activity and creating a *reversible lesion* was the demonstration that such stimulation led to an increase in firing frequency in the nucleus receiving the efferent activity from the nucleus being stimulated (Hashimoto *et al.* 2003), as shown in figure 6.

So, if increasing the frequency of GPi activity through stimulation of the STN had the same effect as burning a hole in the GPi, the reason had to lie in the dynamics of the effect of increasing the firing rate of the GPi. Note two things about the effect of stimulation on the GPi activity in figure 6: (i) the frequency is increased and (ii) the neural response becomes less episodic and more continuous. The rest of this paper seeks to understand and exploit this finding.

Increasingly, we run into examples such as this where you cannot possibly understand the raw data recorded from the nervous system without forming a computational model. The model is necessary to create the equations of motion for the network involved. As with Newton’s laws, it does not take very much of a complex system before even the simplest of nonlinear interactions between elements, whether planets through gravity or neurons through synapses, becomes impossible to put together from visual inspection and guessing.

 Rubin & Terman (2004) set out to ask whether the apparent *stimulation paradox* outlined here was explainable on the basis of the biophysical properties of the neurons within these networks. Their goal was

… to demonstrate, with a computational model, why this is actually not contradictory, but rather is a natural consequence of the properties of the cells involved.

(Rubin & Terman 2004, p. 212)

Fitting empirical models to Parkinson’s data might produce an effective controller, but would be no more insightful than proving that you could fit a model to prove that the neurons did what you had observed them to do. The power of model-based control approaches is that we take the insights from what we have learned about these pieces of brain we are working with, and use those fundamental models to guide our observations and control.

## 8. Reductionist cracking the deep brain stimulation paradox

We will now extend our model, from the oscillations between the GPe and the STN (Terman *et al.* 2002), to the effect of these oscillations on the thalamus through the intermediary way station of the GPi. The motor structures within the thalamus, with apology to the vast complexity of this organ (Guillery & Sherman 2002), will be viewed as a structure whose task is to faithfully relay information. The thalamus will have two inputs: GPi and sensorimotor signals (figure 3).

 Rubin & Terman (2004) distilled the essence of Parkinson’s disease symptoms within the output of the GPi. In the normal state, these cells fire irregularly and do not interfere with thalamic information relay. In Parkinson’s disease, GPi cells fire bursts of action potentials at the frequency of tremor (3–8 Hz). Rubin & Terman (2004) assumed that these burst firing cells will exhibit some degree of synchrony in the pathological state. Their hypothesis is that such clustered firing in bursts would impair the sensorimotor relay properties of the thalamic cells.

The sparse structured network (figure 4*b*) from Terman *et al.* (2002) will be chosen based on the results with the various topologies illustrated in figure 4.

Thalamic cells were modelled with
8.1
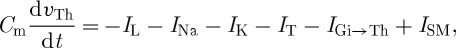

where *I*_Gi→Th_ represents synaptic current from the GPi to thalamus and *I*_SM_ represents sensorimotor input to thalamus. They are of opposite sign because one is inhibitory and the other excitatory.

The specific structure of these currents is of interest to us now, as we will shortly reduce them as a prelude to our control framework.

The leak current is simple,

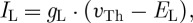

where *g*_L_ is the maximal leak conductance, *v*_Th_ is the transmembrane voltage on the thalamic cell and *E*_L_ is the reversal potential at which there would be no leak current when *v*_Th_=*E*_L_. The sodium current is from Hodgkin & Huxley (1952),

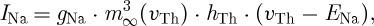

except for the use of Rinzel’s (1985) approximation, substituting 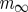 for *m* in the sodium gating variable, and in the following potassium current equation, substituting 1−*h*_Th_ for *n* for the potassium gating variable (*h* is the sodium inactivation gating variable):

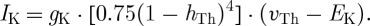

The T-type calcium current equation is
8.2
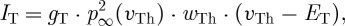

where 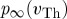 is the T-current gating variable, and *w*_Th_ is the T-current inactivation variable. In these equations, the reversal potentials for leak, sodium, potassium and T-current are *E*_L_, *E*_Na_, *E*_K_ and *E*_T_, respectively. The inactivation variables follow the Hodgkin–Huxley formalism for first-order kinetics,

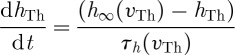

and
8.3
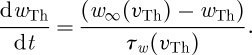

The sensorimotor current, *I*_SM_, was prescribed to be either periodic or at times random. The symmetry introduced in this equation by the cancelling currents −*I*_Gi→Th_+*I*_SM_ will cause us difficulties that we will address in §11 (a more complete discussion of symmetries in reconstruction can be found in Sauer & Schiff 2009).

These thalamocortical (TC) cells are silent if unstimulated. If stimulated with depolarizing current, they fire progressively faster. However, if hyperpolarized, one sees progressively more and more intense rebound activity owing to the T-current.

**Figure 7.**
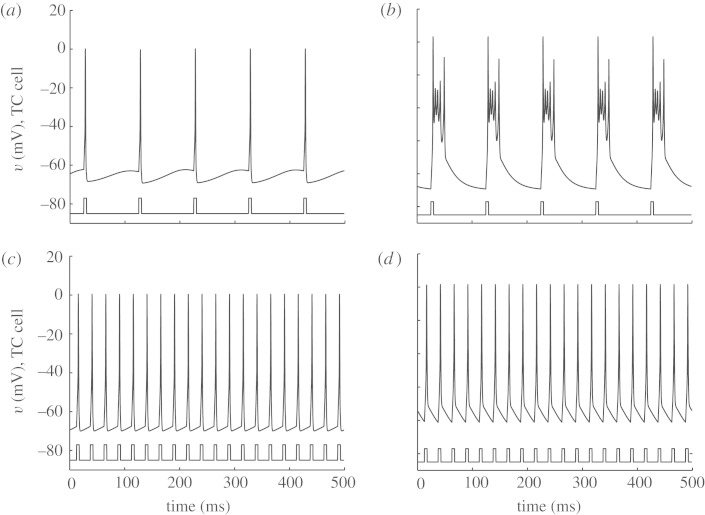
(*a*,*b*) Stimulation of TC-cell model at 10 Hz and (*c*,*d*) 40 Hz, (*a*,*c*) at low and (*b*,*d*) high levels of tonic inhibition. (Adapted from Rubin & Terman (2004).)

We can provide an analogue to sensorimotor stimulation to this TC cell by periodically stimulating it. This is a signal that we hope the cell can relay. In figure 7*a*, we start with slow stimulation. The signal is reliably relayed. Now, simultaneously provide the cell with excessive inhibition, such as in Parkinson’s disease, from an overactive GPi. In figure 7*b*, the baseline membrane potential is now more hyperpolarized. With each sensorimotor pulse, the cell rebound spikes because the T-current is deinactivated. This is because hyperpolarization removes *I*_T_ inactivation, just as in the sodium inactivation in the Hodgkin–Huxley gating variable *h* (Hodgkin & Huxley 1952). But removing inactivation is also relatively slow. In figure 7*b*, there is sufficient time for this variable to be fully deinactivated. Contrast this with higher stimulation frequencies. In figure 7*c*, the TC cell is reliable, and it remains so in the setting of additional inhibition in figure 7*d* because there is insufficient time for the inactivation to deinactivate.

STN cells were modelled with
8.4
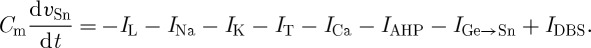

Here, Rubin & Terman (2004) added a high-threshold calcium current, *I*_Ca_, and the synaptic current is now from the GPe to the STN, *I*_Ge→Sn_. There is also a DBS current, *I*_DBS_. The parameters on the STN cells were adjusted so that the cell was spontaneously active, fired at high frequencies when depolarized and had a less prominent rebound than the TC cell. All of these adjustments were to preserve as much of the qualitative distinction between the firing properties of these cells as observed in prior experiments.

GPe cells were modelled with
8.5
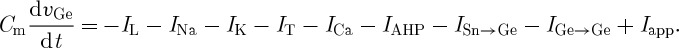

Input from the striatum was modelled with *I*_app_. The GPe cells would continuously fire, initially decrease their firing rate with inhibition, and with more inhibition, cluster fire consistent with results in Terman *et al.* (2002). The GPi cells were similarly modelled, except the parameters were adjusted to account for the experimental evidence that these cells tended to fire faster than GPe cells (DeLong 1971).^2^

**Figure 8.**
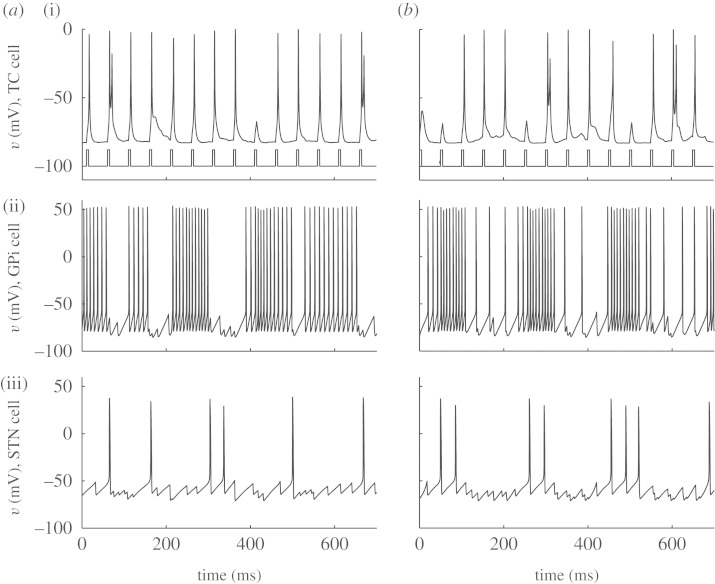
(i) Model of the TC, (ii) GPi and (iii) STN cells to periodic sensorimotor stimulation (i) in the (*a*) normal and (*b*) Parkinsonian states. (Adapted from Rubin & Terman (2004).)

Following the schematic in figure 5, the Parkinsonian state is recreated by increasing the striatal input to the GPe, and decreasing the amount of internal recurrent inhibition within the GPe. The result is that the normal reliability of the TC cell to transmit sensorimotor information, illustrated in figure 8, becomes impaired in the Parkinsonian state. The key quantity here is the error rate of transmitting sensorimotor input into TC spikes. An error index can be created as

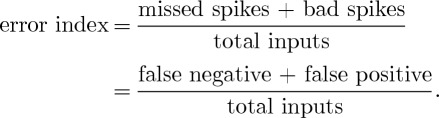

Rubin & Terman (2004) showed that the error rate was significantly elevated in the Parkinsonian^3^ state in comparison with the normal state, and that the error rate could be normalized by simulating DBS using a constant level of high-frequency stimulation of the STN. The key to understand these results is to know what the TC cell is receiving. In the Parkinsonian state, the amount of inhibition is fluctuating more than normal. This induces sequential excess suppression and rebound bursting in the TC cell, which destroys reliability. By applying DBS, the fluctuations that the TC cell receives are decreased, despite an overall increase in GPi-cell firing.

A key insight of Rubin & Terman (2004) was that the qualitative features of the above could be preserved in the TC cell *without* the fast dynamics.^4^ They take their TC-membrane model and remove the fast Hodgkin–Huxley currents for sodium, *I*_Na_, and potassium, *I*_K_, and create a two-variable description of TC-cell-membrane dynamics,
8.6
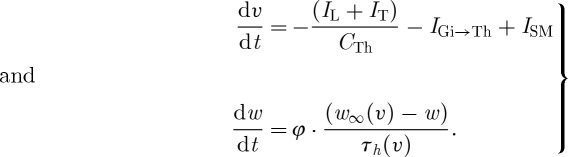

In equations (8.6), *v* represents membrane voltage, *φ* serves as a relative rate constant between the two differential equations, *w* represents T-current inactivation, and the availability of T-current *I*_T_ will be the key to reliability.

The current *I*_Gi→Th_ will be modelled as a true synaptic inhibitory input onto the TC cell as
8.7
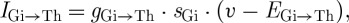

where *s*_Gi_ will be a small positive constant in normal conditions, a periodic square wave in Parkinsonian conditions and a larger positive constant under conditions of DBS. But because there is a reversal potential attached to all synaptic ionic channels, *E*_Gi→Th_, the current *I*_Gi→Th_ will fluctuate with voltage *v*, even when *s*_Gi_ is a constant. The equations for the T-current, *I*_T_, are as given in equations (8.2) and (8.3).^5^

The beauty of the two-variable reduction of the TC cell is that the nullclines can be plotted in two dimensions and visualized,^6^ and further insight into the dynamics gained. It is also a simpler system to fit where one is interested in observing actual data, or developing control laws, than by using the full model.

**Figure 9.**
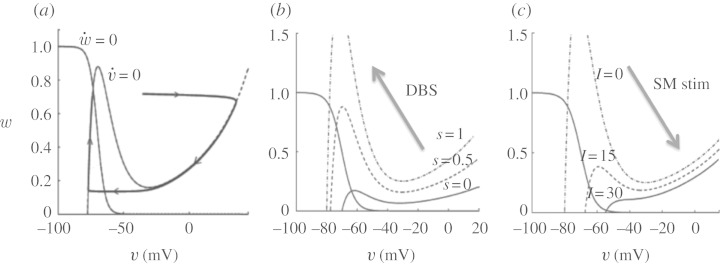
Nullclines for 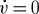 and 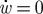. (*a*) The heavy line is a trajectory initiated by depolarizing the rest state at the intersection of the nullclines from −65 to −25 mV, and the subsequent trajectory in the phase plane closely tracks the faster equilibrating *v* nullcline and slowly works its way up along the direction of increasing *w* as the T-current deinactivates (i.e. the availability of T-current, *w*, increases). (*b*) The effect of DBS, elevating the *v* nullcline. (*c*) The effect of sensorimotor stimulation, SM, which lowers the *v* nullcline. (Adapted from Rubin & Terman (2004).)

The nullclines for *v* and *w* are shown in figure 9*a*. In excitatory cells and their models, there is almost always a cubic or N-shaped nullcline for the fast excitatory variable, the voltage *v* in this case. Keep in mind that, in this reduced model, there are no true action potential spikes (no *I*_Na_ or *I*_K_ currents). These phase-space plots show us the slow dance between voltage changes, *v* and *I*_T_ inactivation, *w*. In figure 9*b*, we see the effect of DBS. Increasing the synaptic current from the GPi, *s*_Gi_, as the DBS parameter in equation (8.7) literally adds the *I*_Gi→Th_ current term in equation (8.7) to the solution of the *v* nullcline.^7^ This has a qualitatively opposite effect as increasing excitatory sensorimotor stimulation *I*_SM_ in equation (8.6), which decreases the height of the *v* nullcline. The point where these nullclines intersect is the resting steady state for *v* and for *w*. The T-current inactivation, *w*, is a key factor in whether this system will respond with a rebound burst, respond reliably to a sensorimotor input or be inactive and unreliable by not responding at all.

When the intersection is brought lower on this phase plot, the system requires enough T-current, by having high enough *w*, to be able to push over the peak in the 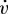 nullcline, the *knee* of this curve (the upgoing bump). As sensorimotor stimulus steadily increases, the value of *w* gradually decreases and the system is set up for trouble.

What this means is that, if the position of the intersection of the nullclines is such that a stimulus (*I*_SM_) comes when the value of *w* is above the knee of the 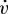 nulcline, then the cell can fire a spike. If the system were subjected to *episodic* bursts of inhibition through a Parkinsonian GPi input to TC, then when the intense inhibition were released the cell would rebound. But hit the cell with an intense enough *steady* DBS input, the cell will maintain sufficient T-current and has no opportunity to rebound (there is no abrupt turnoff of inhibition). Because in the Parkinsonian state, the T-current availability fluctuates on and off, the result is tremor at this fluctuation frequency. DBS can level this out and simultaneously keep the cell in a state ready to fire reliably to excitatory inputs by keeping *w* moderately high.

**Figure 10.**
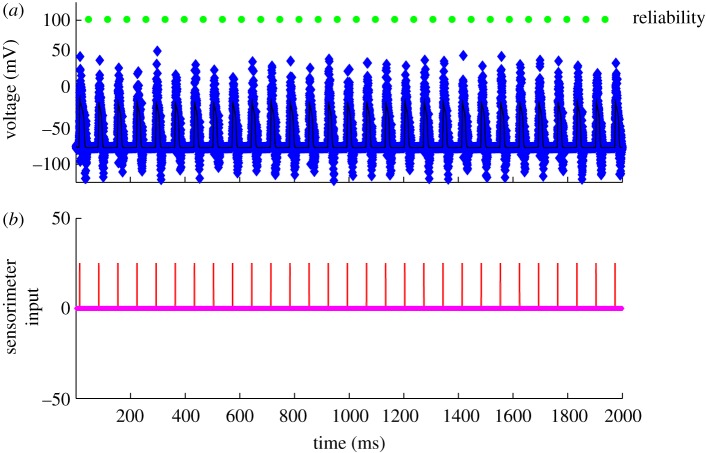
Normal response of the TC cell to periodic sensorimotor stimulation. (*a*) Voltage in reduced TC-cell model (thin black line), with substantial added noise to serve as a noisy observable (blue markers), and (*b*) sensorimotor input (red pulses). Each time a sensorimotor pulse is reliably transmitted, a marker (green) is placed above the successfully transmitted spike. This cell is 100% reliable.

Now let us look deeper into these reduced dynamics in normal, DBS and Parkinsonian states. In figure 10, we see a normal state. The input to the reduced TC cell from the GPi is a modest constant, which represents a state where there is asynchrony among the GPi cells, and there results a modest steady level of inhibition to the TC cells. There is periodic sensorimotor stimulation represented by brief square-wave excitatory inputs (figure 10*b*). A substantial amount of random measurement noise is added to the actual TC voltage. These noisy measurements are what we record from in these models as our *observable* variable. Note that for each sensorimotor stimulus, a ‘spike’ is transmitted from the TC cell. This is reliable transmission (100% reliable in this case).

The DBS state is modelled as increased tonic inhibition on the TC cell. The Parkinsonian case can be idealized as a periodic fluctuation in the GPi input onto the TC cells. In Rubin & Terman (2004), such an effect can be shown as a slow on and off square-wave modulation of GPi output. Using the reduced model, when excess inhibition turns on, *w* gradually increases, T-current becomes more available and spiking becomes more reliable after an initial suppression by the inhibition. When inhibition suddenly stops, *w* remains too high, and an excessive response with a period of rebound excitation occurs. During the trough of the Parkinsonian inhibition fluctuations, spikes are reliably transmitted but *w* gradually inactivates, and when the next inhibitory pulse hits, there is spike failure. This sequence of recurrent spike failure and rebound creates unreliability. Recall that in the reduced model, there are no fast sodium spikes—the slow spikes are periods of increased *excitability*; if we had *I*_Na_ and *I*_K_, we would get a burst of fast sodium spikes riding on the excitability rebound event, and this would also reflect unreliable information transmission. Steady DBS, literally taking out the troughs in the Parkinsonian fluctuations, can restore reliability as we will shortly examine in more detail.

## 9. A cost function for deep brain stimulation

Feng *et al.* (2007*a*,*b*) sought to further explore the work of Rubin & Terman (2004) by introducing *optimization* principles. They, like Rubin & Terman (2004), employed the sparse-structured network of Terman *et al.* (2002).

Why not just use the high-frequency stimulation that presently demonstrates efficacy in clinical use? For one thing, the more energy we apply per day, the sooner the batteries of the stimulators wear out and the devices need to be surgically replaced.

But beyond batteries, as stimulation intensities increase, so will negative cognitive side effects (Alberts *et al.* 2008).^8^ I would pose a general principle that, in the treatment of any dynamical disease of the brain, one needs to optimize the beneficial effects of symptom relief (e.g. tremor), with the inherent effects of increasing cognitive dysfunction with increasing DBS energy.

In addition, it is not trivial at all to make clinical adjustments to a patient’s stimulation parameters. Once set, the parameter space of stimulation intensity and frequency, along with duty cycle, is enormous. Making changes, waiting several days or weeks to see the steady-state effect and trying to optimize settings are difficult in patients. So, if a stimulator is working and showing benefit, optimizing in this ad hoc fashion is generally avoided. Nevertheless, there is evidence that there are patients whose symptoms can be improved at frequencies different from the standard starting frequency of 130 Hz (Moreau *et al.* 2008). And who is to say that a more complex non-periodic stimulus delivered in open loop might not be more beneficial than the standard periodic ones?

Lastly, the beneficial effect of DBS over the long term in a patient with a neurodegenerative disease (and perhaps those without degeneration) is a moving target. Patients with DBS treatment of Parkinson’s disease continue to deteriorate within the tempo of progression of Parkinson’s disease (Krack *et al.* 2003). Neural networks learn and change in response to stimulation. A means to automatically adapt and optimize such stimulation over time will be a very valuable advance in our stimulation technology.

One requires metrics for optimization. Feng *et al.* introduced a *reliability* measure similar to Rubin & Terman (2004), as well as a GPi-cell *correlation* metric. Neither metric has a clear route to practical application in human subjects using our present optimization done *by hand*. But these tools can be immediately applied if we construct a customized computational model for an average or specific patient. And this model-based approach that can be employed in future automated closed-loop optimization.

The structure of their cost function was (Feng *et al.* 2007*a*)

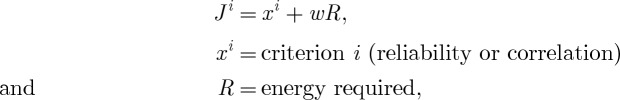

where *w* is a weighting parameter. They employed a genetic algorithm to search their parameter space and accomplish optimization.

One way of implementing such a cost function is to integrate the current coming out of the stimulator, *I*_DBS_, and subtract this weighted integral from the reliability, Rel, as

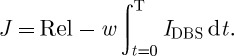



**Figure 11.**
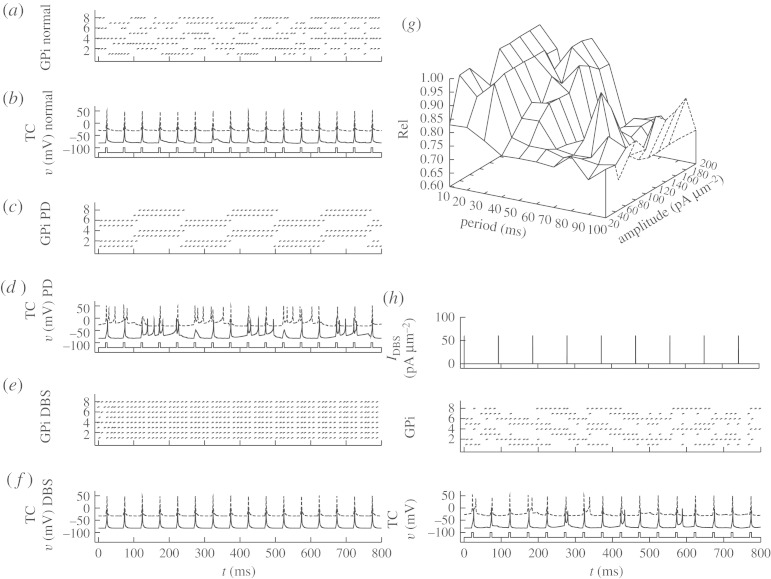
Exploration of the parameter space of period and amplitude focusing on reliability. (*a*,*b*) Normal; (*c*,*d*) Parkinson’s disease (PD); and (*e*,*f*) DBS in PD. (*g*) Optimization of reliability (Rel) as a function of period and amplitude. (*h*) Slower DBS frequency at 80 ms reliability peak. (Adapted from Feng *et al.* (2007*a*).)

So, starting from a sparse structured network, with two TC cells, they replicate the fundamental findings of Rubin & Terman (2004) in figure 11*a*–*f*.

They then use their genetic algorithm to explore the parameter space of frequency and amplitude as shown in figure 11*g*. In the far rear corner of figure 11*g* lies the high-frequency and high-amplitude parameter regime typically employed in clinical use, and the reliability there is high. But notice the other peaks. The most prominent secondary peak is between 40 and 50 Hz, and indeed there is some clinical evidence that this frequency range might be therapeutic (Moreau *et al.* 2008). In figure 11*h*, they show the results of a minor peak at a period of 80 ms (12.5 Hz); there is actually some clinical study of stimulation in the range of 5–20 Hz, which was (unfortunately) singularly unimpressive (Eusebio *et al.* 2008). We note that another exploration of frequency in an extended model of Rubin & Terman (2004) demonstrated that the frequencies greater than 100 Hz were most effective (Pirini *et al.* 2008).

Reliability is an interesting measure. There seems no way to directly measure, in real life, the quantities needed to calculate reliability: sensorimotor inputs and thalamic relay cell output in response to this input. But such inputs can be readily modelled using a data assimilation framework. Feng *et al.* (2007*a*) also employ a correlation measure, which was a combination of autocorrelation and cross-correlation among GPi cells. This would require a micro-electrode array to be placed within the GPi. Such micro-array technology is now becoming available on the shafts of clinical DBS electrodes. A simpler way to infer such correlation in practice would be to estimate spectral concentration in GPi local field potentials, and this is directly observable from an ordinary DBS electrode placed within the GPi.

An important contribution of Feng *et al.* (2007*a*) is that they demonstrated in this computational model that both stochastic stimulation and complex waveforms can demonstrate potential efficacy.

## 10. Fusing experimental globus pallidus recordings with deep brain stimulation models

A creative next step was performed by Guo *et al.* (2008). They took recordings of GPi spike timings from normal and MPTP primates, with and without DBS. They applied a structure function to these spike timings to re-create an estimate of the synaptic currents produced in the thalamic relay cells from such spiking. They used the model from Rubin & Terman (2004) to estimate the reliability of the transmission of spikes through the thalamus. Their results demonstrate that the error index (described above) is higher for the MPTP monkeys without DBS or with subtherapeutic DBS, and consistently lowest for normal monkeys and those MPTP monkeys with therapeutic levels of DBS.

This is another example of how we can apply models in real-life applications to Parkinson’s disease treatment. One would require the recording of an array of GPi cells. One could then follow the prescription of Guo *et al.* (2008) to estimate ongoing reliability, and potentially perform closed-loop optimization.

A very interesting insight from Guo *et al.* (2008) was to *reverse correlate* what the inhibitory current drive from the GPi was *prior* to a spike. Spikes can be faithful in following a sensorimotor input, can miss their chance to follow or multiple bad spikes can ensue. Guo *et al.* (2008) found that missed spikes correspond to a relatively rapid rise in inhibition, and bad spikes correspond to a relatively rapid decrease in inhibition. This is exactly what one might have expected from the reduced model effects illustrated in figure 9.

## 11. Towards a control framework for Parkinson’s disease

Parkinson’s disease will be the first *dynamical disease* (Mackey & Glass 1977) amenable to management through model-based feedback control systems.

First, examine the schematic in figure 3. We can now relatively safely place electrodes in the STN, GPi or thalamus.^9^

What about the small amount of damage that we get from just passing a 1–2 mm thick depth electrode? Recall that in Parkinson’s disease, making a lesion in these small structures is clinically beneficial. Amazingly, we observe that in the course of placing DBS electrodes in Parkinson’s disease patients, there is an immediate clinical improvement in about *half* of patients at the conclusion of electrode placement, *before* the electrode is connected to the stimulator (Tasker 1998). This has been termed the *micro-thalamotomy effect* (Tasker 1998). A nearly identical experience has been reported in the placement of DBS electrodes in small thalamic targets for epileptic seizure suppression (Hodaie *et al.* 2002). Our DBS treatments are probably a combination of small lesions overlain with chronic electrical stimulations.

Despite the beneficial effect of some of the microlesions created by electrode insertion, the best clinical strategy is almost certainly to keep the number of electrodes inserted to the absolute minimum.^10^ But the network that is important in Parkinson’s disease control is spread out over at least four separate structures: the STN, GPe, GPi and thalamus.

From a clinical perspective, the GPi would probably be an easier and safer target than the smaller STN or thalamic targets. A small haemorrhage or lesion effect in the GPi has a reasonable chance of actually benefitting some of the Parkinson’s disease symptoms. But with a superb animal model in the MPTP primates, exploring which nuclei are optimal to record data from, and work out the control algorithms, offers a valuable way to learn how to build such systems prior to their implantation in human patients. Nevertheless, given that our clinical opportunities with routine implantation in patients are ongoing, the chance to take advantage of existing STN or GPi human placements in algorithm development is highly attractive.

The models we have just discussed permit us to sample from a single nucleus within this network and reconstruct what the remainder of the network is doing. Our present state-of-the-art models appear sophisticated enough to consider using these models in a data assimilation framework. And the technology to perform chronic real-time sampling from these structures is presently available and in clinical use.

So let us begin to outline a control theoretical framework from what we have discussed above.

The first issue with parameter estimation for equation (8.6) is to notice that there is a symmetry with the terms (−*I*_Gi→Th_+*I*_SM_). The tracking algorithm will simply apportion such current equally between these two sources. One option is to combine the currents into one sum, which physically is what is happening to the TC cells. But this defeats our purpose here, where we would like to estimate quantities such as (−*I*_Gi→Th_) in isolation. A more extensive discussion of symmetries in such equations can be found in Sauer & Schiff (2009).

**Figure 12.**
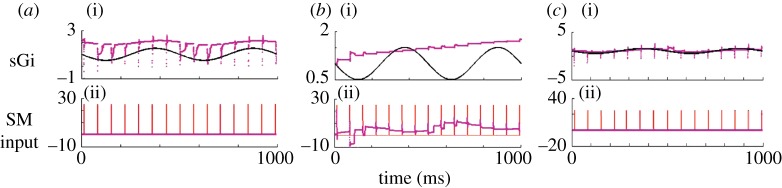
Tracking and estimating parameters as a function of process noises *Q*. Estimates of synaptic current from GPi (i, sGi) and sensorimotor input (ii, SM input). Process noise parameters are (*a*) sGi : *Q*=30, SM : *Q*= 0.01, (*b*) sGi : *Q*=0.01, SM : *Q*=30 and (*c*) sGi : *Q*=10.0, SM : *Q*=0.01. The algorithmic incorporation of such process noise can be explored in the code archive with Schiff & Sauer (2008).

When faced with such symmetries, it is best to get rid of them (Sauer & Schiff 2009). If that is not feasible, then an empirical rule of thumb seems to be to set process noise in rough proportions to the average magnitudes of the corresponding variables. One could adaptively tune these process noises over time by tracking innovation error.^11^ Process noise, *Q*, is uncertainty commonly added to the model of the process (the *plant* in control jargon) in analogous applications. In this particular instance of ensemble Kalman filtering, we will use several *Q* values as the assumed variance in the respective parameters to be tracked. In addition to apportioning variance to the respective parameters, this also has the benefit of preventing a Kalman filter from driving the parameter covariance to zero.^12^ In figure 12*a*, the *Q* for (−*I*_Gi→Th_) is 30, while the *Q* for *I*_SM_ is 0.01. Note that the rhythmicity of (−*I*_Gi→Th_) is resolved, while none of the features of *I*_SM_ are picked up (figure 12*a*). Now reverse the situation, setting *Q* for (−*I*_Gi→Th_) to 0.01 and the *Q* for *I*_SM_ to 30; the (−*I*_Gi→Th_) will be poorly tracked, but the sensorimotor inputs *I*_SM_ are better tracked (figure 12*b*). A more balanced set of process noises, where *Q* for (−*I*_Gi→Th_) is 10, and *Q* for *I*_SM_ is 0.01, yields a more optimal tracking of (−*I*_Gi→Th_).

We can calculate a running *reliability* index over the past 10 ms (ignoring for now having to objectively define ‘bad’ spikes unrelated to sensorimotor inputs) as

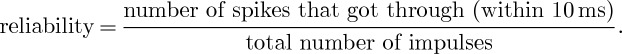



Let us now focus on the heart of the dynamics critical for the Parkinsonian state—the T-current inactivation *w*. This is a rather independent variable, in time scale and with respect to symmetry, and lies at the heart of the issues of unreliability.

**Figure 13.**
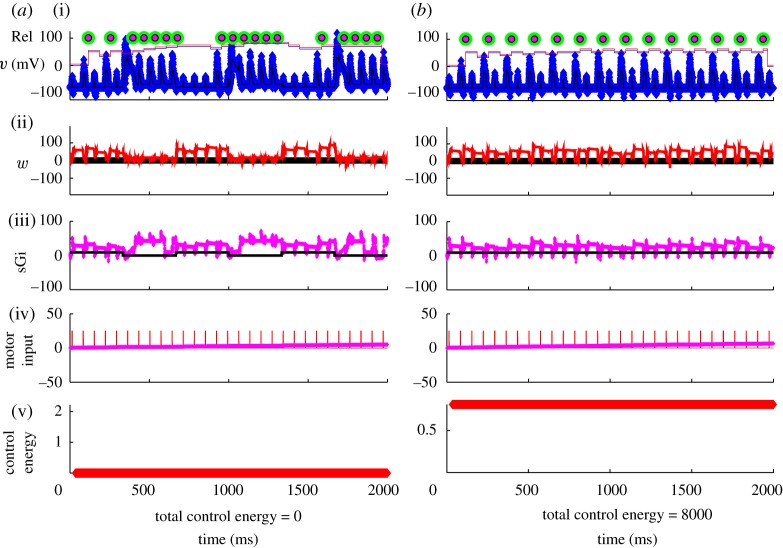
(*a*) Uncontrolled reduced TC-cell dynamics in the Parkinsonian state with fluctuating current from the GPi (sGi). (*b*) Perfect DBS stimulation filling in the troughs in the fluctuating current from the GPi. (i) Noisy observable voltage (blue symbols), reliability as piecewise continuous plots without (blue) and with (red) control on, the green circles are the timing of SM spikes and the smaller red circles are transmitted spikes. (ii) Estimated *w* (red). (iii) Real (black) and estimated (magenta) synaptic current from the GPi (sGi, estimated values multiplied by 10 for discriminability from the true values). (iv) Real (red) and estimated (magenta) motor input (we are deliberately not trying to reconstruct motor input in the reconstruction through *Q* ratio adjustment). (v) Running control energy, the squared value of the control signal at each time point and the total sum of squares given as total control energy.

In figure 13*a*, I employ the reduced TC-cell model from Rubin & Terman (2004) in a data assimilation framework. The observable will be noisy voltage from the TC cell in figure 13*a*(i),*b*(i). Below that is shown the reconstructed estimated T-current inactivation, *w*, and the reconstruction of estimated GPi and sensorimotor activity ((ii)–(iv), respectively). The techniques used to accomplish this reconstruction are detailed in Schiff & Sauer (2008), Sauer & Schiff (2009) and Ullah & Schiff (2009), and a code archive for the basics of such data assimilation can be found in the electronic supplementary material with Schiff & Sauer (2008).

One could similarly record the average GPi output from a DBS electrode in GPi and reconstruct the estimated TC-cell activity.

The sensorimotor input, in the figure 13*a*(iv),*b*(iv), causes the model TC cell to generate or fail to generate reliable spike activity in the figure 13*a*(i),*b*(i), I build up a reliability index by averaging the last 10 sensorimotor spike responses, and plot this at the very top of the figure 13*a*(i),*b*(i).

**Figure 14.**
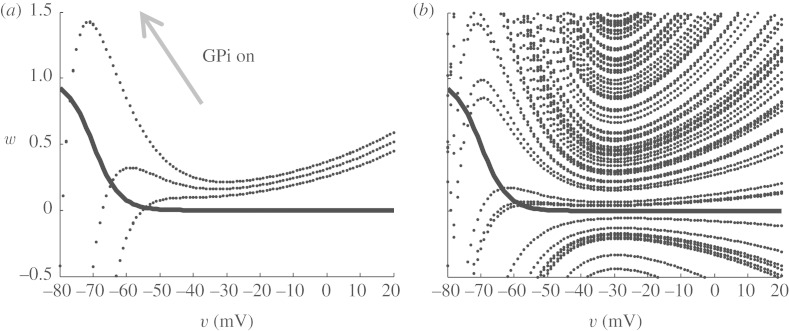
Nullclines for *w* (thick line) and *v* (dotted line). (*a*) With increased GPi inhibition onto the reduced TC cell, the *v* nullcline is elevated with respect to the *w* nullcline. (*b*) Reconstruction estimates of the nullclines from noisy measurements.

It is tempting to examine and consider control from the nullclines. There is an extensive literature in control theory on what is generally termed *variable structure control*. First described by Utkin in the 1970s (Utkin 1977), this control law strategy is now more commonly referred to as *sliding-mode* control (DeCarlo *et al.* 1988; Young *et al.* 1999). We could use such nullclines to generate control functions that we seek to target the system towards. In figure 14*a*, we see the actual nullcline intersection of our simulation of the reduced TC cell as the GPi current switches from off to on (refer to figure 9). The *w* nullcline does not change here as the GPi input fluctuates. In figure 14*b*, I show an estimation of these nullclines from a reconstruction of these curves using a UKF. There is a great deal of uncertainty in the *v* nullcline. So, estimating a control surface in this phase space is not a trivial problem that we know how to solve at present.

But recall that, as discussed by Rubin & Terman (2004), it is not the nullcline intersection, so much as the value of *w*, that is the most valuable single feature that helps explain the dynamical response of the TC cell. So, let us estimate *w* by itself.

In the second figure 13*a*(ii) is the estimate of the reconstructed T-current availability *w* (red line). Note that *w* increases when the GPi current increases in figure 13*a*(iii) (solid black line). I have deliberately adjusted the ratios of the process noises so that the sensorimotor input is *not* tracked well (figure 13*a*(iv)), in order to minimize the symmetry of this current in the voltage equation. Figure 13*a*(v),*b*(v) shows the running plot of control energy, which is zero for this example without feedback.

Now, let us use *perfect* DBS. We will, in the sense of Rubin & Terman (2004), fill in the gaps of the fluctuating synaptic current from the GPi, and show these results in figure 13*b*. Providing a steady level of DBS increases the amount of inhibition arriving on the TC cell. This has the effect of removing the fluctuations in inhibition creating the gaps in reliability shown in figure 13*a*, but it also dampens down the responsiveness of the TC cell. Only about half of the incident sensorimotor spikes get reliably through. And the cost in terms of control energy is high (8000 on an arbitrary scale). On the other hand, the fluctuations in *w* are reduced, and we maintain an overall high level of *w* (figure 13*b*(v)).

**Figure 15.**
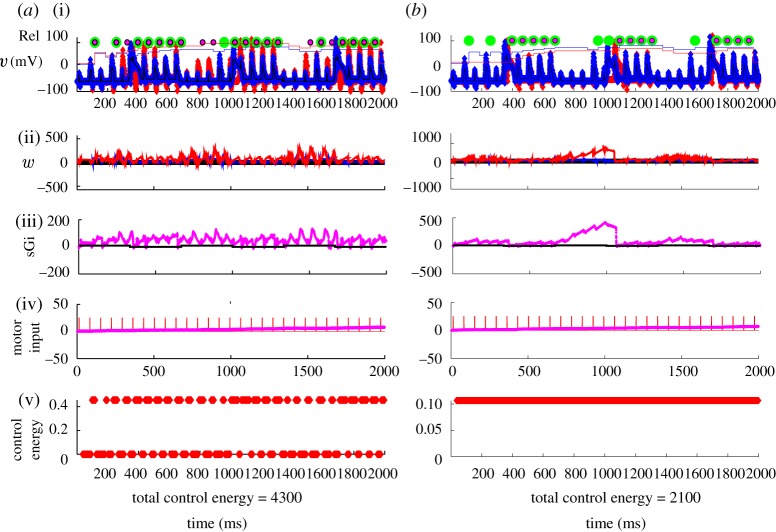
(*a*) Feedback control scenario based on turning on and off DBS based on a running average of the estimated T-current availability. (*b*) A scenario where a constant amount of DBS is simply added to the fluctuating Parkinsonian GPi output. No adjustment of GPi constant stimulation comes close to the reliability achieved with the closed-loop feedback scenario shown in (*a*). Symbols as in figure 13.

Now, let us use adaptive feedback based upon the estimated *w*. In figure 15*a*, the effect of an optimal amount of proportional feedback gain based on a moving average (35 ms) of the estimate of *w* is shown. This feedback is very effective in restoring most of the unreliable (missing) spikes. The total cost in control energy is about half of the perfect DBS case shown in figure 13*b*. Now, let us present a more realistic DBS scenario than in figure 13*b*, one in which a constant stimulation (open loop) will be *added* to the fluctuating Parkinsonian GPi signal. In figure 15*b*, the largest (and most effective) additive current that is stable in this model is shown. We are here limited by the relatively large peak fluctuating GPi currents being applied already in the Parkinsonian state, as the dynamics of the TC cell become unstable if the impinging currents become excessive. The figure shows that such constant DBS does not appear capable of achieving the reliability possible with feedback control.^13^

**Figure 16.**
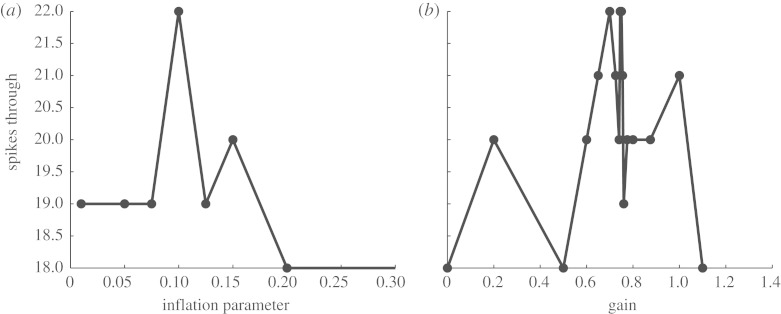
(*a*) Optimizing reliability as spikes that are transmitted reliably through the TC cell as a function of the covariance inflation parameter. (*b*) Proportional control gain parameter.

It is important to note that in this simple scenario, there is a range of additional adjustable parameters which are important. First, there is the ever present issue of covariance inflation (Anderson & Anderson 1999).^14^ In figure 16*a*, we see that adjusting the small covariance inflation parameter has a substantial effect on the reliability of the adaptive system. Similarly, the gain on the feedback control is important. In figure 16*b*, we see that optimizing gain readily reveals a region where spike throughput is best. Both of these functions are not smooth, and implementing such an algorithm should be done with continual adaptation of such parameters based upon the monitoring of the system performance.

**Figure 17.**
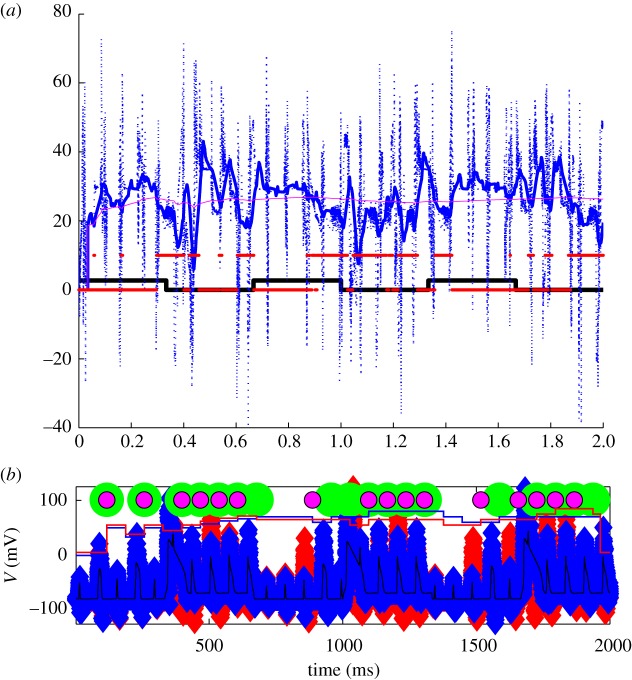
(*a*) Estimated GPi input to TC cell (blue dotted line), and smoothed short-term moving average of this GPi input (blue solid line). We take a long-term moving average of this current (magenta line) as an adapting threshold to tell when the more instantaneous GPi input is fluctuating up or down. Crossing below the threshold determines when to turn the control on (red). The actual GPi fluctuations are shown (black lines). (*b*) The results with control off (blue markers) and on (red markers), and the uncontrolled (green markers) and controlled (magenta markers) spikes transmitted are shown. The running reliability of the TC cell is plotted as a piecewise continuous line for uncontrolled (blue line) and controlled (red line) scenarios.

Another alternative is to generate a control signal based upon the estimated GPi output, shown in figure 17. As we know the control signal added, we can subtract this to follow just the underlying estimated GPi input to the thalamus. As the estimates of GPi input to thalamus are noisy (dotted blue line in figure 17), it is helpful to create a moving average filter of this estimate (solid blue line) to prevent the controller from turning on and off too often. As at the heart of Parkinson’s disease physiology are the large-scale slower fluctuations, we can create a long-term running average of the GPi output, much longer than the noise reducing short-term moving average, and let this serve as an adapting threshold (magenta line). Control is turned on whenever the short-term moving average (blue solid line) falls below the long-term moving average (magenta line). The control is applied in this case by turning on the stimulator with the same constant amplitude, shown as the red lines. The underlying true GPi output fluctuations are shown as a black line for comparison. Note that a *control reliability* can be calculated as the fraction of time that the control signal (red) is on or off in correct reflection of the peaks and valleys in the true GPi signal (black). In this example, the control reliability is 67 per cent. But the effect on the neuronal reliability in the TC cell, our goal, is not very impressive (there are a few additional transmitted spikes in the controlled case, but also some missed spikes).

**Figure 18.**
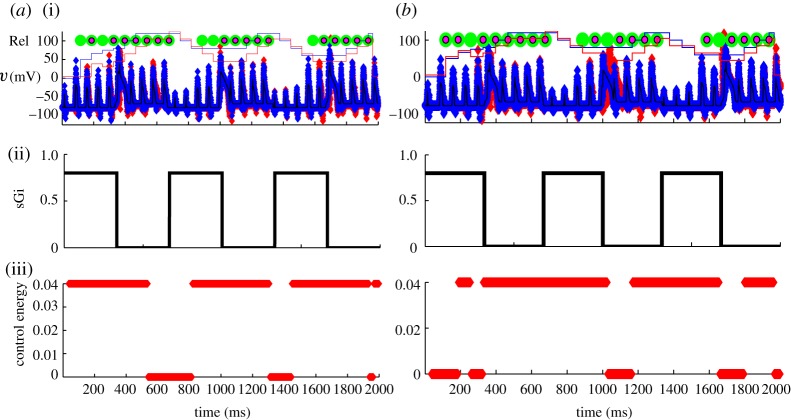
Control of TC-reduced-cell model using reliability as a control parameter. (*a*) A threshold of turning on GPi stimulation when reliability less than 0.9 is shown. (*b*) A different strategy, using an inverse approach is shown. In (*b*) control is turned on when reliability is greater than 0.5. Note that the relevant reliability in both examples is the controlled (red) piecewise continuous line in (i) (the blue reliability line is the uncontrolled state shown for comparison). The inherent delays in employing the moving average of reliability can be exploited so that inverse reliability control can be more reliable than using a more intuitive strategy based on turning on stimulation when reliability falls.

As reliability is our goal, and as we are estimating it, why not use it as the control parameter? Recognize that the way I have been calculating TC-cell reliability in figures 14–17 employed a moving average of relatively infrequent events (the incoming spikes are on a time scale significantly slower than membrane dynamics such as *w*). So, this formulation of reliability is substantially delayed with respect to the dynamics of the system. This delay creates the type of results seen in figure 18*a* for control based upon turning the stimulator on when estimated reliability is too low.

On the other hand, what is delayed for the past is predictive of the future. If you use low reliability as a control variable, by the time the moving average of reliability decreases to an arbitrary threshold, turning on stimulation will probably come too late. But for periodic fluctuations, for which Parkinson’s disease abounds, you can invert your control variable. This uses *high* reliability as a control threshold. By the time reliability becomes high enough, the lack of inhibition will be drawing to a close, and the incipient upturn in inhibition will be imminent. Such results can be interesting, as shown in the improved control in figure 18*b*. In these examples attempting to use estimated reliability as a control variable, the control reliability with respect to GPi output never rose above 46–50%. On the other hand, our goal is to treat thalamic reliability, not the waveform of GPi output, and calculating control through running estimates of thalamic reliability is something we are presently exploring in more complete models.

To summarize this section, we have explored a variety of dynamics for controlling the thalamic cells. These examples envision direct stimulation of GPi, and our results are consistent with those of Pirini *et al.* (2008) demonstrating that open-loop stimulation of GPi can be a strong suppressor of thalamic activity (figure 13). Control through estimation of nullclines is not a trivial problem (figure 14), but we have not approached this with the addition of nullcline constraints and inertia that would further improve this strategy. It is interesting that a direct estimation of the synaptic currents bombarding the thalamus (figure 17) was not as effective as an estimation of the T-current activation (figure 15) in these proportional control algorithms. Similarly, control from reliability estimates (figure 18) was not as effective as the estimation of T-current activation. One reason why T-current may have been so effective is that the time constant on the T-current activation equation is intermediate between the rapid estimation of GPi input currents and the relatively slow reliability estimates. This intermediate time constant may help damp out the relatively high levels of noise that we have imposed on this system, but still offer suitable responsiveness for an essential component of the pathological dynamics. Whether more instantaneous estimates of reliability would serve to improve this situation is unclear (the reliability uncertainty would increase with less averaging). Nevertheless, in principle, and in full model simulations (not shown), reliability is a compelling control variable at the heart of the control problem in Parkinson’s disease. Lastly, regardless of method chosen, adaptively adjusting covariance inflation and control gain (figure 16) are fundamental necessities to optimize such algorithms over time.

## 12. Looking foward

I have here just touched upon the potential strategies for the use of computational models in Parkinson’s disease control. For purposes of illustration, I have used an idealized thalamic cell to gain intuition for what is possible in designing future device controllers.

Sketching out the unstudied issues is worthwhile. The calculations in figures 14–18 assume that there are two electrodes inserted—a recording electrode in the thalamus and a stimulating electrode in the GPi or STN. The ideal Parkinson’s controller would work off a single electrode, albeit one with multiple contacts, inserted into just one nucleus. We can employ separate contacts for recording and stimulation along the electrode shaft. Perhaps, picking up the oscillatory rhythms from the GPi or STN would be sufficient for feedback control of those same nuclei. On the other hand, the models presented here give us the freedom to take such oscillatory dynamics from the GPi or STN and estimate the reliability of the thalamus. We might never be able to record a good estimate of sensorimotor input to the actual thalamus, but we can provide such signals, or a range of such signals, to a model thalamus that is functioning as an observer model system when only the GPi or STN serves as the recording site.

The reduced model of the TC cell is valuable for gaining intuition. Adding back the fast Hodgkin–Huxley sodium and potassium currents may be helpful in using such a model to track the thalamic dynamics, as might using an ensemble of such cells. But one of the appeals of such reduced models is that they can represent ensemble activity. Using a scaled-up version of this reduced TC cell, *renormalized* to represent an ensemble of such cells, might well be an effective way to track thalamic dynamics in real brains.

We are presently using the full model of Rubin & Terman (2004) to explore setting up a control system by working with the more complete computational Parkinsonian brain. All of the same principles mentioned above for the reduced model tracking are applicable in the full model. But one of the interesting facets of contrasting full and reduced models is that, beyond the obvious computational overhead of computing additional variables and parameters, the need to fit these additional variables and parameters may render more complete models *less accurate* in tracking complex systems than by employing more reduced models. This seems counterintuitive, but the use of reduced models is commonplace in meteorological forecasting in place of the full atmospheric dynamical equations (Kalnay 2003), and in recent fluid dynamic experiments, reduced models have also proven their use in such data assimilation frameworks (Cornick *et al.* 2009). An important feature of the use of such reduced models in tracking scenarios is that the optimal parameters in these *inadequate* models are often non-physical (Cornick *et al.* 2009; Sauer & Schiff 2009). But what matters for Parkinson’s disease patients is that we optimize our controllers and improve symptoms better than with open-loop approaches, not that we reverse engineer their failing basal ganglia. Model validation is not our primary goal in neural system control.

**Figure 19.**
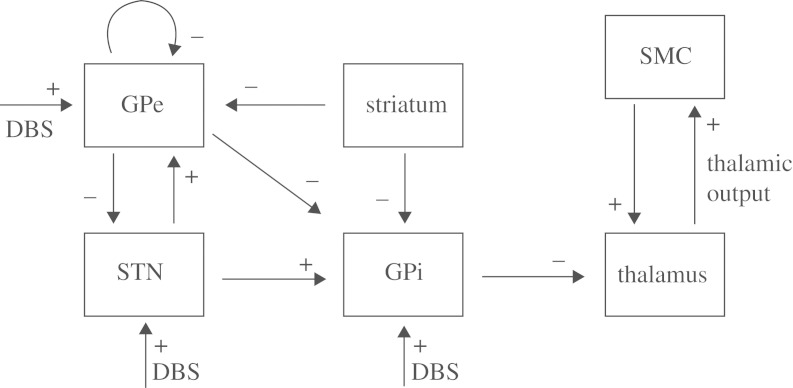
The extended Rubin–Terman model as proposed by Pirini *et al.* (2008). (Adapted from Pirini *et al.* (2008).)

Recent work has extended the model of Rubin & Terman (2004) to take into account more biologically relevant connections, the *direct* versus *indirect* pathways, from striatum to the structures of the basal ganglia (figure 19). The further incorporation of relevant model components may well give greater fidelity to the realistic basal ganglia dynamics in Parkinson’s disease. But with our goal being control, such fidelity through complexity will need to be balanced against accuracy of data assimilation and control metrics. One of the important issues raised by Pirini *et al.* (2008) is that a more complex model of the thalamic cell’s function, beyond the simple relay, is probably important. One example of this is *action-selection* theory, which envisions that the basal ganglia serves to select from competing neuronal efforts for access to the final common path of motor movement (Humphries *et al.* 2006). Furthermore, as in human patient experience, the DBS target sites are not equivalent. Indeed, maintaining the flexibility to perform model-based control the STN, GPi or VIM, depending upon a patient’s symptom complex, is a challenge for future work.

Parkinson’s disease will probably be the first dynamical human disease where model-based control principles will show efficacy. The bar is not really that high. Were we merely to demonstrate that we can significantly extend the battery life of implanted stimulators, the control algorithms will be valuable. But as I hope the above calculations allude to, such device longevity may well be accompanied by improved performance. The parameter space that we presently face clinically in adjusting a patient’s stimulator is, for all practical purposes, infinite. Adaptive algorithms that can continually optimize stimulator performance will give our present empirical trial and error efforts very good competition. So long as our devices operate within the electrical safety limits, and incorporate present DBS protocols as alternatives if the adaptive ones are not better, our patients have nothing to lose and improved lives to gain.

## Footnotes

1 This is done by image- and micro-electrode-guided stereotactic placement of a thin probe to the target nucleus within the brain, and the use of a calibrated radio-frequency thermal lesion.

2 Some further discussion about the applicability of DeLong (1971) to the classification of human Parkinsonian cellular activity can be found in Schiff *et al.* (2005).

3 It is semantically sensible to designate dynamics similar to those seen in Parkinson’s disease as ‘Parkinsonian’, and to similarly term analogous models as Parkinsonian.

4 This is only one example of a case where slow excitability dynamics may be more important than the faster spiking dynamics in determining pathological activities in dynamical disease. In recent work, we have shown that, in models of epileptic seizures, relatively slow fluctuations in potassium dynamics may underlie the more complex spike dynamics seen in individual cells (Cressman *et al.* 2009; Ullah *et al.* 2009).

5 The square on  can be dropped in the reduced model. See Rubin & Terman (2004) appendix for all equation details.

6 Nullclines are the curves where the rate of change of the variables is equal to zero. The intersection of more than one nullcline gives us the solutions, stable or unstable, of the system variables.

7 The reason is that when d*v*/d*t*=0, the solution of *v* iswhere the very high positive reversal potential for calcium, *E*_Ca_, always makes (*v*−*E*_Ca_) negative. Because of the negative reversal potential *E*_Gi→Th_ for the inhibitory synapses, (*v*−*E*_Gi→Th_) is almost always positive in equation (8.7). So, the net effect is that increasing *I*_Gi→Th_ leads to an increase in the *v* nullcline.

8 There are recent reports that steady low-amplitude DC stimulation to some parts of the brain might improve cognitive capabilities in certain paradigms (Marshall *et al.* 2006). But this is a polarization effect on certain parts of the cortex. To the author’s knowledge, all pulse stimulation of deep brain structures will have negative effects on cognitive information processing as stimulation is increased. This will probably apply to DBS treatment of cognitive disorders, such as depression and obsessive compulsive disorder, as well as to Parkinson’s disease.

9 The best current data, from A. Benabid (2007, personal communication), suggests an 8 per cent risk of a complication per depth electrode pass. Half of these events (such as small haemorrhages) are asymptomatic. Half of the remaining give transient effects. A reasonable estimate is that 2 per cent of electrode passes into the brain using modern stereotactic techniques will result in a significant neurological complication (Lozano *et al.* 2009).

10 This is a conjecture by the author, and one can argue in the abstract that we are accumulating a tremendous body of data showing that microlesions plus electrical stimulation through single electrodes is efficacious. But we have no such body of data suggesting that multiple microlesions in separate unilateral structures will be beneficial. And from a surgical standpoint, every electrode insertion increases operative time, increases the risk of haemorrhage and neurological deficit and increases the risk of infection. Surgical minimalism should be a guiding principle in the absence of evidence strongly supporting the contrary.

11 Innovation errors are the differences between what your model predicts you should observe in the next measurement, and what is actually measured.

12 A detailed discussion of process noise can be found in Simon (2006).

13 In real life, such model instability would not arise. So long as we stay within the safety limit of the electrical stimulation, we might be able to deliver more GPi current to the TC cell. But the reliability steadily decreases in this scenario as this type of additive DBS increases in the model, as too much inhibition shuts off the TC cells. We find no inference that such open-loop stimulation is going to be as effective as an intelligent adaptive approach as illustrated in figure 15*a*.

14 In fact, Julier & Uhlmann (1997*a*) had an adjustable inflation parameter that they required to tune their original unscented filter. As in so many of these recursive filter designs, there is a trade-off between tracking accuracy versus stability with greater uncertainty. One also needs to counter the tendency of these filters to recursively drive the uncertainty to zero, which ruins tracking ability.
